# Upregulation of RSPO2-GPR48/LGR4 signaling in papillary thyroid carcinoma contributes to tumor progression

**DOI:** 10.18632/oncotarget.22692

**Published:** 2017-11-25

**Authors:** Yea Eun Kang, Jin-Man Kim, Koon Soon Kim, Joon Young Chang, Mingyu Jung, Junguee Lee, Shinae Yi, Hyeon Woo Kim, Jung Tae Kim, Kyungmin Lee, Min Jeong Choi, Seul Ki Kang, Seong Eun Lee, Hyon-Seung Yi, Bon Seok Koo, Minho Shong

**Affiliations:** ^1^ Department of Endocrinology and Metabolism, College of Medicine, Chungnam National University, Daejeon 35015, South Korea; ^2^ Department of Medical Science, College of Medicine, Chungnam National University, Daejeon 35015, Republic of Korea; ^3^ Department of Pathology, College of Medicine, Chungnam National University, Daejeon 35015, Republic of Korea; ^4^ Department of Pathology, Daejeon St. Mary’s Hospital, College of Medicine, The Catholic University of Korea, Daejeon 34943, Republic of Korea; ^5^ Department of Otolaryngology-Head and Neck Surgery, College of Medicine, Chungnam National University, Daejeon 35015, Republic of Korea

**Keywords:** RSPO2, GPR48/LGR4, papillary thyroid carcinoma, BRAF^V600E^ mutation, β-catenin pathway

## Abstract

The signaling pathway involving the R-spondins and its cognate receptor, GPR48/LGR4, is crucial in development and carcinogenesis. However, the functional implications of the R-spondin-GPR48/LGR4 pathway in thyroid remain to be identified. The aim of this study was to investigate the role of R-spondin-GPR48/LGR4 signaling in papillary thyroid carcinomas. We retrospectively reviewed a total of 214 patients who underwent total thyroidectomy and cervical lymph node dissection for papillary thyroid carcinoma. The role of GPR48/LGR4 in proliferation and migration was examined in thyroid cancer cell lines. R-spondin 2, and GPR48/LGR4 were expressed at significantly higher levels in thyroid cancer than in normal controls. Elevated GPR48/LGR4 expression was significantly associated with tumor size (P=0.049), lymph node metastasis (P=0.004), recurrence (P=0.037), and the BRAFV600E mutation (P=0.003). Moreover, high GPR48/LGR4 expression was an independent risk factor for lymph node metastasis (P=0.027) and the BRAFV600E mutation (P=0.009). *in vitro* assays demonstrated that elevated expression of GPR48/LGR4 promoted proliferation and migration of thyroid cancer cells, whereas downregulation of GPR48/LGR4 decreased proliferation and migration by inhibition of the β-catenin pathway. Moreover, treatment of thyroid cancer cells with exogenous R-spondin 2 induced activation of the β-catenin pathway through GPR48/LGR4. The R-spondin 2-GPR48/LGR4 signaling axis also induced the phosphorylation of ERK, as well as phosphorylation of LRP6 and serine 9 of GSK3β. Our findings demonstrate that upregulation of the R-spondin 2-GPR48/LGR4 pathway contributes to tumor aggressiveness in papillary thyroid carcinoma by promoting ERK phosphorylation, suggesting that this pathway represents a novel therapeutic target for treatment of differentiated thyroid cancer.

## INTRODUCTION

G-protein coupled receptors (GPCRs), including thyrotropin receptor, are important in both normal thyrocytes and differentiated thyroid cancer cells [1]. Recently, a group of GPCRs named leucine-rich-repeat-containing G-protein-coupled receptors (LGRs) were studied as regulators of both organogenesis and carcinogenesis [2–4]. GPR48/LGR4 is related to LGR5 and LGR6, with approximately 50% amino acid identity among the three proteins, and is widely expressed in multiple tissues from the early stages of embryogenesis to adulthood [5, 6]. In a previous study, endogenous GPR48/LGR4 expression was monitored by gene reporter assays in transgenic mice: the protein was localized to the Rathke’s pouch in E14 mouse embryos, and expressed in some cells at the border of follicles in the adult mouse thyroid [7]. The GPR48/LGR4 mRNA is also present in normal human thyroid tissue, as revealed by Northern blot analysis, but relatively few studies have investigated the role of GPR48/LGR4 in human thyroid diseases [5, 6].

Recent studies show that the R-spondin (RSPO) family, a group of four newly identified secreted proteins, promotes Wnt/β-catenin signaling. The Wnt/β-catenin is essential in embryonic development as well as in self-renewal and maintenance of adult stem cells. Their biological effects are mediated by high-affinity binding to LGR receptors and sequential induction of LRP6 phosphorylation [8–11]. Lack of RSPO2 signaling in the embryonic stage results in thyroid malformation [12, 13]. These findings showed that the RSPO signaling axis is indispensable for normal development of the thyroid gland in animal models, but the expression status and roles of RSPO–GPR48/LGR4 signaling components in human thyroid gland remain to be elucidated.

However, several lines of evidence indicate that GPR48/LGR4 participates in carcinogenesis. Elevated GPR48/LGR4 expression in cancer tissue is significantly correlated with regional metastasis, and upregulation of GPR48/LGR4 promotes cell proliferation in multiple cancers by stimulating Wnt/β-catenin signaling [14, 15]. Upregulation of GPR48/LGR4 expression, associated with downregulation of P27^kip1^, promotes invasiveness and metastasis in colorectal carcinoma cells [16]. In addition, GPR48/LGR4 contributes to tumor metastasis by stimulating β-catenin/TCF signaling, and is associated with poor prognosis in colorectal cancer [17]. GPR48/LGR4 is also overexpressed in prostate cancer, concomitant with activation of the PI3K/AKT signaling pathway [18]. Thus, GPR48/LGR4 signaling may promote tumorigenesis by potentiating Wnt/β-catenin activity, which is elevated in thyroid cancer [19, 20].

Based on this premise, we investigated the RSPO–GPR48/LGR4 signaling axis in normal thyroid gland and papillary thyroid carcinomas (PTCs). We observed that RSPO2 and its receptor, GPR48/LGR4, were expressed in normal thyroid gland and at higher levels in in PTCs, particularly in patients with regional tumor progression, including lymph node (LN) metastasis. In addition, elevated expression of GPR48/LGR4 promoted proliferation and migration of thyroid cancer cells, whereas downregulation of GPR48/LGR4 decreased proliferation and migration by inhibiting the β-catenin pathway. Collectively, our results indicate that upregulation of the RSPO2–GPR48/LGR4 signaling axis promotes tumor aggressiveness in PTCs.

## RESULTS

### Expression of GPR48/LGR4 in human normal thyroid gland and thyroid carcinomas

**Figure 1:**
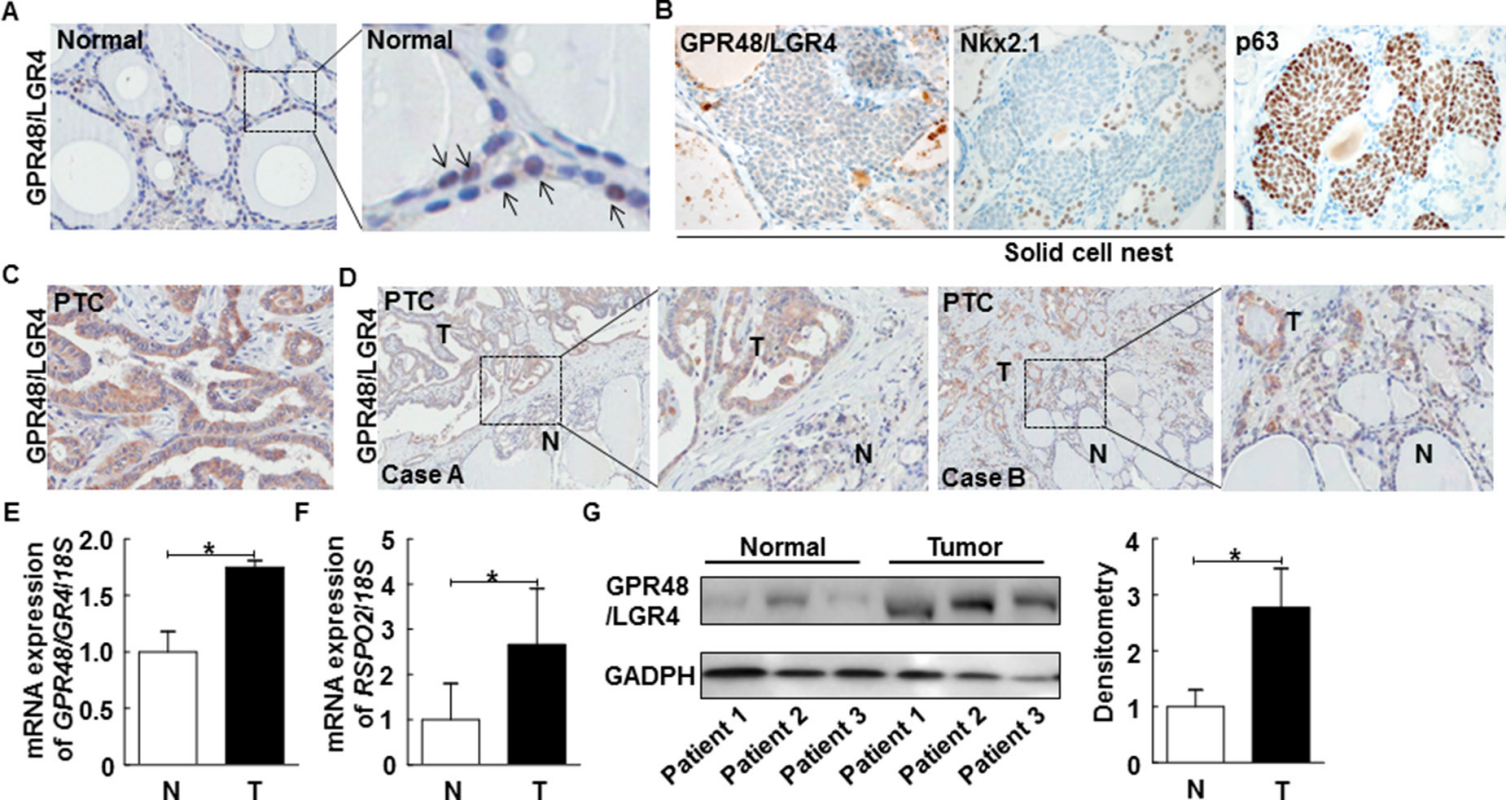
Representative expression of GPR48/LGR4 in human normal thyroid and thyroid carcinomas **(A)** Representative images of normal human thyroid gland immunostained for GPR48/LGR4 (brown) and counterstained with hematoxylin, along with 4× enlarged images. Arrows indicate GPR48/LGR4-positive follicular cells. Magnification: 200× and 800×. **(B)** Representative images of SCNs in human thyroid gland immunostained for GPR48/LGR4, Nkx2.1, and p63. Magnification: 200×. **(C)** Representative photos of human PTCs immunostained for GPR48/LGR4 (brown) and counterstained with hematoxylin. Magnification: 200×. **(D)** Representative expression of GPR48/LGR4 in tumors and corresponding normal thyroid tissue of PTCs, along with 4× enlarged images. Magnification: 100×. N, normal tissue; T, tumor tissue. **(E)** *GPR48*/*LGR4* mRNA levels in total cell lysates acquired from paired clinical specimens of normal (N) and tumor (T) tissues, examined by RT-PCR of specimens from six patients with PTC. **(F)** *RSPO2* mRNA levels in total cell lysates acquired from paired clinical specimens of normal (N) and tumor (T) tissues, examined by RT-PCR of specimens from six patients with PTC. **(G)** Representative images of immunoblot analysis for detection of GPR48/LGR4 in paired clinical specimens of normal (N) and tumor (T) tissues. ^*^, *P*<0.05.

First, we sought to measure the expression of GPR48/LGR4 in normal human thyroid gland. For this purpose, we obtained normal thyroid tissue from contralateral lobes of thyroid glands removed during surgery for differentiated thyroid cancer. GPR48/LGR4 was weakly positive in the nucleus and cytoplasm of normal follicular epithelium (Figure 1A). The solid cell nests (SCNs) of the human thyroid are ultimobranchial body remnants that may be the origin of pluripotent cells contributing to folliculogenesis, as well as formation of some thyroid tumors [21, 22]. LGRs, which have recently attracted attention as markers of adult stem cells, are important for maintenance of stem cell functions. To determine whether GPR48/LGR4 represents a marker of a specific population of SCNs, we observed GPR48/LGR4 immunoreactivity in SCNs. As shown in Figure 1B, cells forming SCNs did not exhibit significant immunoreactivity against either Nkx2.1 or GPR48/LGR4, although p63, an epithelial stem cell marker, exhibited consistently higher expression in SCNs. Thus, GPR48/LGR4 is only expressed in normal thyroid follicular epithelium, but is rarely expressed in embryonic remnants.

In PTC, the expression pattern of GPR48/LGR4 differed from that in normal thyroid gland. Most tumor cells in PTCs were GPR48/LGR4-positive, and the staining was more intense than in normal follicular cells positive for this marker (Figure 1C). Staining of GPR48/LGR4 was distributed in the membrane and cytoplasm, but not the nucleus. Additionally, we compared the intensity and distribution of GPR48/LGR4 staining in normal thyroid and tumor tissues of patients with PTC (Figure 1D). Interestingly, invasive fronts of tumor tissues exhibited high levels of GRP48/LGR4 staining, and GPR48/LGR4 was more intensely and extensively expressed in tumor lesions than in normal tissue (Figure 1D). To investigate the expression of *GPR48/LGR4* mRNA in human PTC, we analyzed paired clinical specimens of tumor and non-tumor tissues from six patients with PTC by RT-PCR. The results revealed that expression of *GPR48/LGR4* mRNA was ∼1.75-fold higher in PTC tissues than in normal tissues (Figure 1E; *P*<0.05). Moreover, expression of the mRNA encoding RSPO2, a secreted Wnt agonist that is one of the endogenous ligands of GPR48/LGR4, was ∼2.6-fold higher than in normal tissues (Figure 1F; *P*<0.05). Expression of the mRNA encoding *RSPO3*, another secreted Wnt agonist and endogenous ligand of GPR48/LGR4, did not differ significantly between tumor and normal tissues (data was not shown). Expression of *RSPO1* and *RSPO4* mRNA was not detectable in thyroid tissues. These data suggest that GPR48/LGR4 and its ligand RSPO2 are overexpressed in human PTC. We evaluated protein expression in lysates from normal and PTC tissue specimens to confirm the differential expression of GPR48/LGR4 between tumor and normal tissues. As shown in Figure 1G, GPR48/LGR4 was detectable in both tumors and normal tissue, but expression was more intense in tumors.

### Correlation between immunoreactivity of GPR48/LGR4 and clinicopathologic features of patients with differentiated thyroid carcinoma

Next, to evaluate the relationship between GPR48/LGR4 immunoreactivity and clinicopathologic features of PTC, we first characterized clinicopathologic features of 214 patients treated for PTC at Chungnam National University Hospital from 2003 to 2010. Clinicopathologic characteristics of enrolled patients are provided in Table 1. The mean age of the study subjects was 48.4 years (range, 22–84), the majority of the patients were female (81.8%), and the average size of the primary tumor was 1.1 ± 0.8 mm. The proportion of patients with multicentricity was 41.6% (89/214); capsular invasion, 75.7% (162/214); extra-thyroid extension, 70.1% (150/214); and lymphovascular invasion, 79.9% (171/214). Of the 214 PTC patients, 63.1% (135/214) had LN metastases and 19.2% (41/214) had lateral neck LN metastases. Recurrence was observed in 7.5% (16/214) of patients, and the mean follow-up period was 46.1 ± 22.9 months. GPR48/LGR4 immunoreactivity was detected in 97.7% (209/214) of cases. To quantify immunoreactivity of GPR48/LGR4 and BRAF^V600E^ in PTC, we adopted a scoring system that combined the intensity and the distribution of positive staining: 0, no staining; +1, weak staining in focal tumor areas; +2, moderate staining in most tumors; and +3, strong staining in most tumors. Representative images of GPR48/LGR4 staining and BRAF^V600E^ staining are shown in Supplementary Figure 1A and 1B. The distribution of the GPR48/LGR4 staining was as follows: no staining in 5 cases (2.4%); weak staining (+1) in 15 (20%); moderate staining (+2) in 42 (19.6%); and strong staining (+3) in 152 (71.0%). The distribution of BRAF^V600E^ staining was as follows: no staining in 34 cases (15.9%); and positive staining [i.e., presence of BRAF^V600E^ including weak staining (+1), moderated staining (+2), and strong staining (+3)] in 180 (84.1%). Evaluation of the 16 patients with recurrence revealed recurrent disease, as determined by ultrasonography in 16 (100%), by PET-CT in 13 (81.3 %), and by neck CT in 4 (25%). All 16 patients exhibited locoregional recurrence (15 local and 1 locoregional recurrence), confirmed by US-guided FNAB of neck LN. All 16 patients underwent salvage surgery and exhibited no evidence of recurrence at the last follow-up.

**Table 1:** Clinicopathologic parameters of patients

Variables		Mean ± SD or number of patients (%)
Age, years		48.4 ± 12.9
Gender	Male	39 (18.2)
	Female	175 (81.8)
Tumor size	≤ 1cm	85 (39.7)
	> 1cm	129 (60.3)
Multicentricity	No	125 (58.4)
	Yes	89 (41.6)
Microscopic capsular invasion	No	52 (24.3)
	Yes	162 (75.7)
Extrathryoid extension	No	64 (29.9)
	Yes	150 (70.1)
Lymphovascular invasion	No	43 (20.1)
	Yes	171 (79.9)
Lymph node metastasis	No	79 (36.9)
	Yes	135 (63.1)
Central lymph node metastasis	No	79 (36.9)
	Yes	135 (63.1)
Lateral lymph node metastasis	No	173 (80.8)
	Yes	41 (19.2)
Locoregional recurrence	No	198 (92.5)
	Yes	16 (7.5)
BRAF^V600E^ mutation	No	34 (15.9)
	Yes	180 (84.1)
Follow-up period (months)		46.1 ± 22.9

Next, we analyzed the relationship between the clinicopathologic parameters and expression of GPR48/LGR4 in PTCs. For this, the patients were divided into two groups according to GPR48/LGR4 immunoreactivity. In univariate analysis, high expression of GPR48/LGR4 was significantly associated with several clinicopathologic parameters including tumor size (*P*=0.050), LN metastasis (*P*=0.004), locoregional recurrence (*P*=0.037), and presence of BRAF^V600E^ (*P*=0.003) (Table 2). To determine the role of GPR48/LGR4 as an independent determinant of aggressive phenotypes in PTC, multivariate analysis using stepwise logistic regression was conducted on parameters revealed to be significant by the univariate analysis. In the multivariate analysis, high expression of GPR48/LGR4 was an independent risk factor of LN metastasis (*P*=0.027, OR, 3.156) and the presence of BRAF^V600E^ (*P*=0.009, OR, 4.557) (Table 3). Thus, GPR48/LGR4 positivity was highly associated with markers of tumor aggressiveness.

**Table 2:** Relationship between intensity of GPR48/LGR4 staining and clinicopathologic factors in 214 patients

Variables		No. of patients	LGR4		
			Low	High	*P* value
Age, years	< 45≥ 45	81133	2537	5696	0.634
Gender	MaleFemale	39175	1052	29123	0.612
Tumor size	≤ 1cm>1cm	85129	3131	5498	0.050^*^
Multicentricity	NoYes	12589	3527	9062	0.710
Microscopic capsular invasion	NoYes	52162	1547	37115	0.982
Extrathryoid extension	NoYes	64150	1943	45107	0.880
Lymphovascular invasion	NoYes	43171	1547	28124	0.339
Lymph node metastasis	NoYes	79135	3230	47105	0.004^*^
Central lymph node metastasis	NoYes	79135	3230	47105	0.004^*^
Lateral lymph node metastasis	NoYes	17341	557	11834	0.062
Locoregional recurrence	NoYes	19816	611	13715	0.037^*^
BRAF^V600E^ mutation	NoYes	34180	1745	17135	0.003^*^

^*^P< 0.05 between the two categories for a given variable.

**Table 3:** Multivariate analysis of the relationship between GPR48/LGR4 expression and clinicopathologic factors

Factors	Exp(β)	SE	95.0% CI	*P value*
Tumor size>1cm	1.384	0.329	(0.726, 2.641)	0.323
Lymph node metastasis	3.156	0.520	(1.139, 8.747)	0.027^*^
Locoregional recurrence	3.826	1.066	(0.474, 4.918)	0.208
BRAF^V600E^ mutation	4.557	0.579	(1.466, 14.162)	0.009^*^

SE, standard error; Exp(β), odds ratio; CI, confidence interval.

### Effects of GPR48/LGR4 on thyroid tumor cell growth and migration

**Figure 2:**
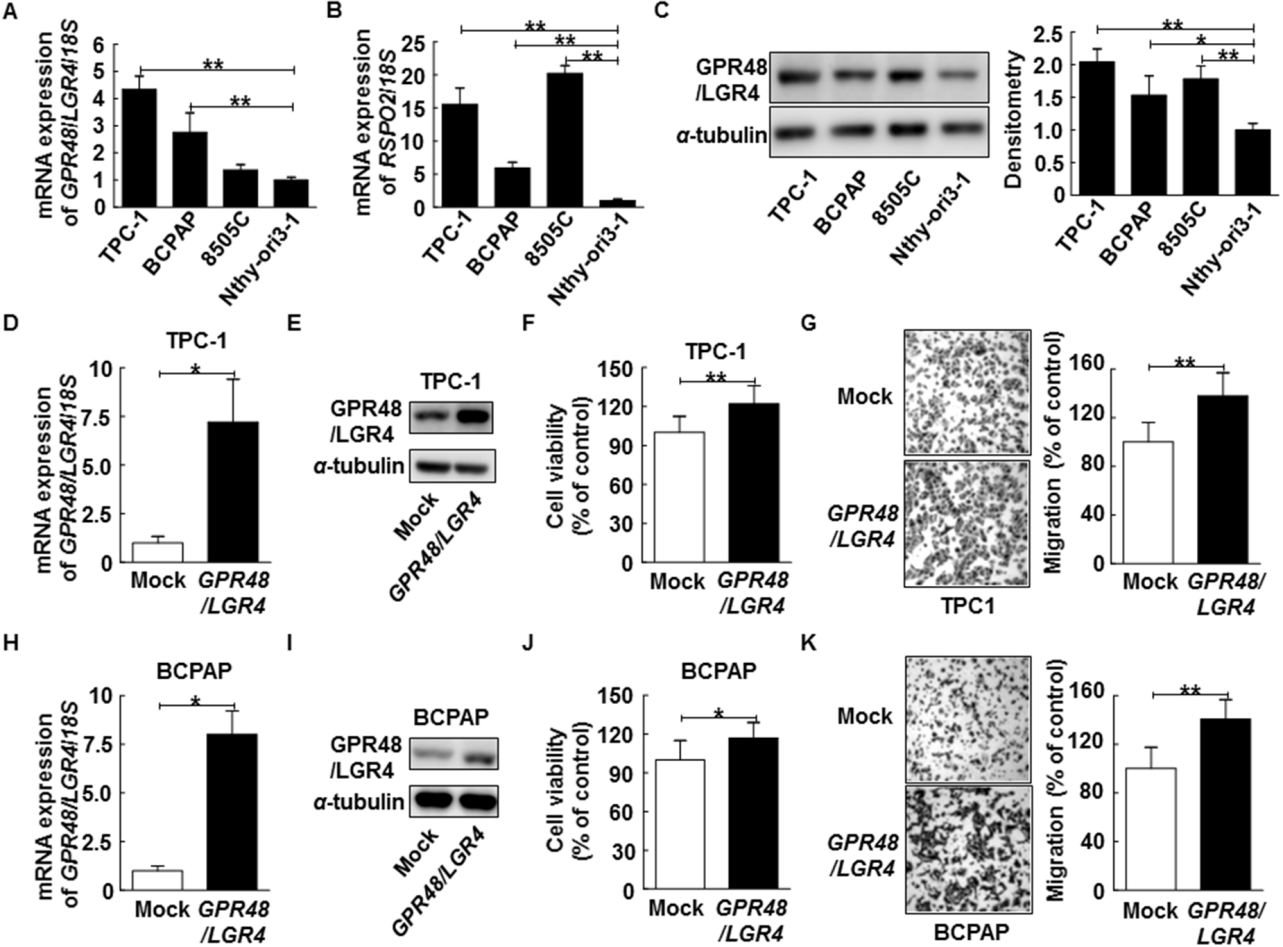
Effect of GPR48/LGR4 on viability and migration of differentiated thyroid carcinoma cells **(A)** *GPR48*/*LGR4* mRNA levels in thyroid carcinoma cell lines. **(B)** *RSPO2* mRNA levels in thyroid carcinoma cell lines. **(C)** Representative images of immunoblot analysis for detection of GPR48/LGR4 in thyroid carcinoma cell lines. **(D)** TPC-1 cells were transiently transfected with *GPR48*/*LGR4* or mock cDNA. **(E)** Representative images of immunoblot analysis for detection of of GPR48/LGR4 in TPC-1 cells transfected with *GPR48*/*LGR4* or mock cDNA. **(F)** Effect of GPR48/LGR4 on cell viability in TPC-1 cells transfected with *GPR48*/*LGR4* cDNA or empty vector, as determined by CCK-8 assay. **(G)** Effect of GPR48/LGR4 on migration in TPC-1 cells transfected with *GPR48*/*LGR4* cDNA or empty vector, as determined by Transwell chamber assay. **(H)** BCPAP cells transiently were transfected with *GPR48*/*LGR4* or mock cDNA. **(I)** Representative images of immunoblot analysis for detection of GPR48/LGR4 in BCPAP cells transfected with *GPR48*/*LGR4* or mock cDNA. **(J)** Effect of GPR48/LGR4 on cell viability of BCPAP cells transfected with *GPR48*/*LGR4* cDNA or empty vector, as determined by CCK-8 assay. **(K)** Effect of GPR48/LGR4 on migration in BCPAP cells transfected with *GPR48*/*LGR4* cDNA or empty vector, as determined by Transwell chamber assay. Each figure is representative of three independent experiments performed in triplicate. ‘Mock’ indicates cells that were transfected with an empty vector. ^*^, *P*<0.05; ^**^, *P*<0.01.

Given the elevated expression of GPR48/LGR4 in thyroid cancer tissue, we investigated the role of GPR48/LGR4 in proliferation and migration of thyroid cancer cells. We found that *GPR48/LGR4* mRNA expression was markedly higher in the thyroid cancer cell lines TPC-1, BCPAP, and 8505C than in Nthy-ori3-1, a normal thyroid follicular cell line derived from a human adult (Figure 2A). In parallel experiments, we observed that *RSPO2* expression was also higher in the thyroid cancer cell lines (*P*<0.001) (Figure 2B). Protein expression of GPR48/LGR4, as determined by Western blot analysis, was also higher in the thyroid cancer cell lines than in Nthy-ori3-1 (Figure 2C). To assess the effect of GPR48/LGR4 in tumorigenesis of differentiated thyroid carcinoma, we transfected a GPR48/LGR4 expression plasmid into TPC-1 and BCPAP, and investigated growth by CCK-8 viability assay and migration by Transwell assay (Figure 2D-2K). GPR48/LGR4-overexpressing TPC-1 and BCPAP exhibited significantly elevated cell viability (122% and 117%, respectively) (Figure 2F and 2J). Additionally, the number of migrated cells significantly increased in GPR48/LGR4-overexpressing TPC-1 and BCPAP (138% and 140.5%, respectively) compared with control (Figure 2G and 2K).

### Inhibition of GPR48/LGR4 blocks thyroid cancer cell proliferation and migration

**Figure 3:**
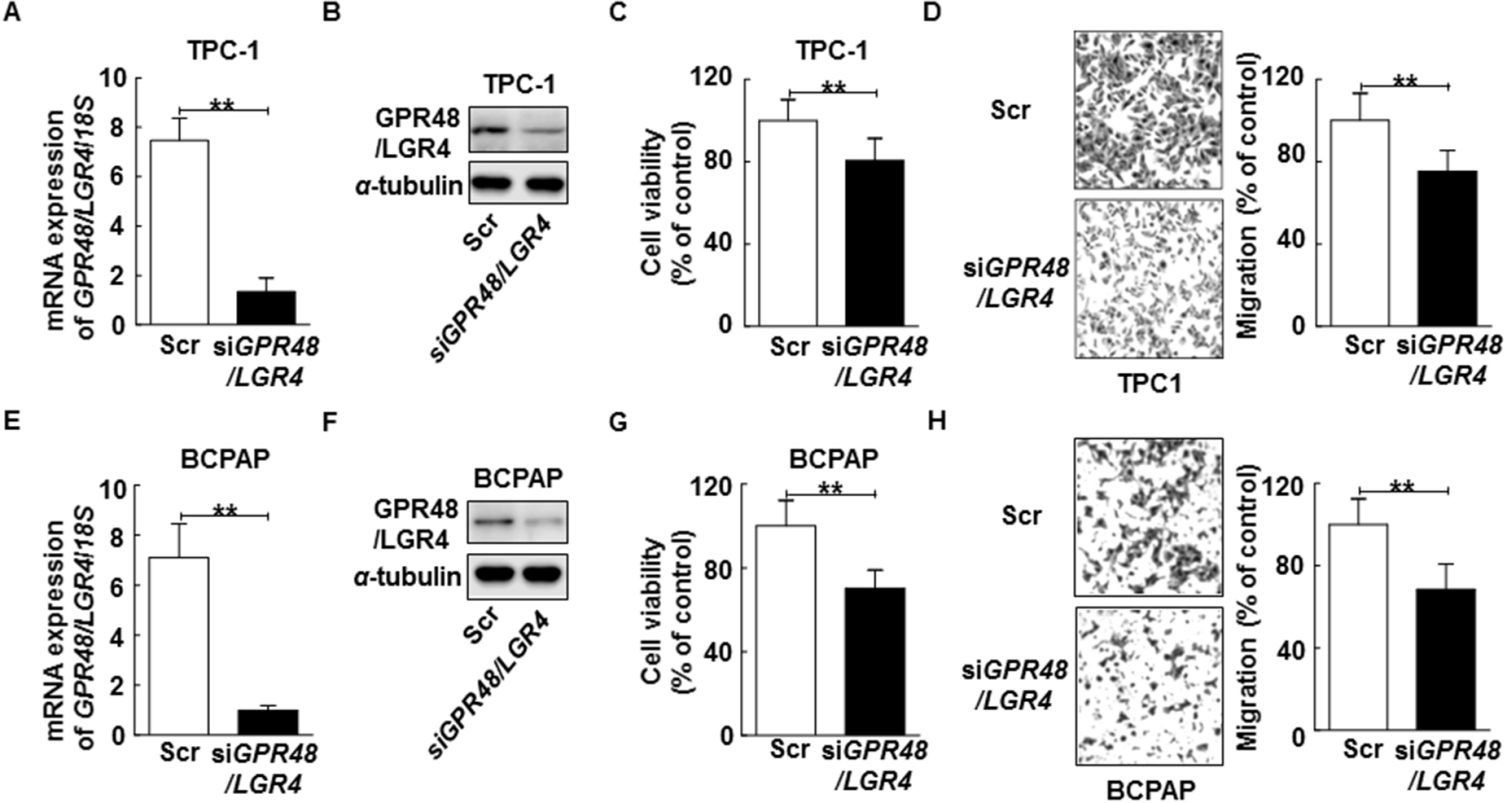
Inhibition of GPR48/LGR4 decreases proliferation and migration of differentiated thyroid carcinoma cells **(A)** TPC-1 cells transiently transfected with *GPR48*/*LGR4*-specific or scrambled siRNA. **(B)** Representative images of immunoblot analysis for detection of GPR48/LGR4 in TPC-1 cells transfected with *GPR48*/*LGR4*-specific or scrambled siRNA. **(C)** Effect of GPR48/LGR4 on cell viability in TPC-1 cells transfected with *GPR48*/*LGR4*-specific or scrambled siRNA, as determined by CCK-8 assay. **(D)** Effect of GPR48/LGR4 on migration in TPC-1 cells transfected with *GPR48*/*LGR4*-specific or scrambled siRNA, as determined by Transwell chamber assay. **(E)** BCPAP cells were transiently transfected with *GPR48*/*LGR4*-specific or scrambled siRNA. **(F)** Representative images of immunoblot analysis for detection of GPR48/LGR4 in BCPAP cells transfected with *GPR48*/*LGR4*-specific or scrambled siRNA. **(G)** Effect of GPR48/LGR4 on cell viability in BCPAP cells transfected with *GPR48*/*LGR4*-specific or scrambled siRNA, as determined by CCK-8 assay. **(H)** Effect of GPR48/LGR4 in BCPAP cells transfected with *GPR48*/*LGR4*-specific or scrambled siRNA, as determined by Transwell chamber assay. ‘Scr’ indicates cells that were transfected with a scrambled siRNA. ^*^, *P*<0.05; ^**^, *P*<0.01.

To evaluate the effects of GPR48/LGR4 on cancer tumorigenesis, we transfected cells with small interfering RNA oligos (siRNA) targeting *GPR48/LGR4* or with scrambled siRNA. Treatment with the targeting siRNA led to a significant reduction of expression of *GPR48/LGR4* in both TPC-1 and BCPAP cells (Figure 3A, 3B, 3E and 3F). *GPR48/LGR4* knockdown significantly decreased the cell viability of TPC-1 and BCPAP (80.5% and 85.2%, respectively) (Figure 3C and 3G). Furthermore, knockdown of *GPR48/LGR4* significantly decreased the migration of TPC-1 and BCPAP (75.2% and 68.5%, respectively), whereas the scrambled siRNA control had no such effect (Figure 3D and 3H).

### GPR48/LGR4 knockdown in thyroid cancer cells downregulates the β-catenin pathway by preventing activation of MAPK/ERK1/2 signaling

**Figure 4:**
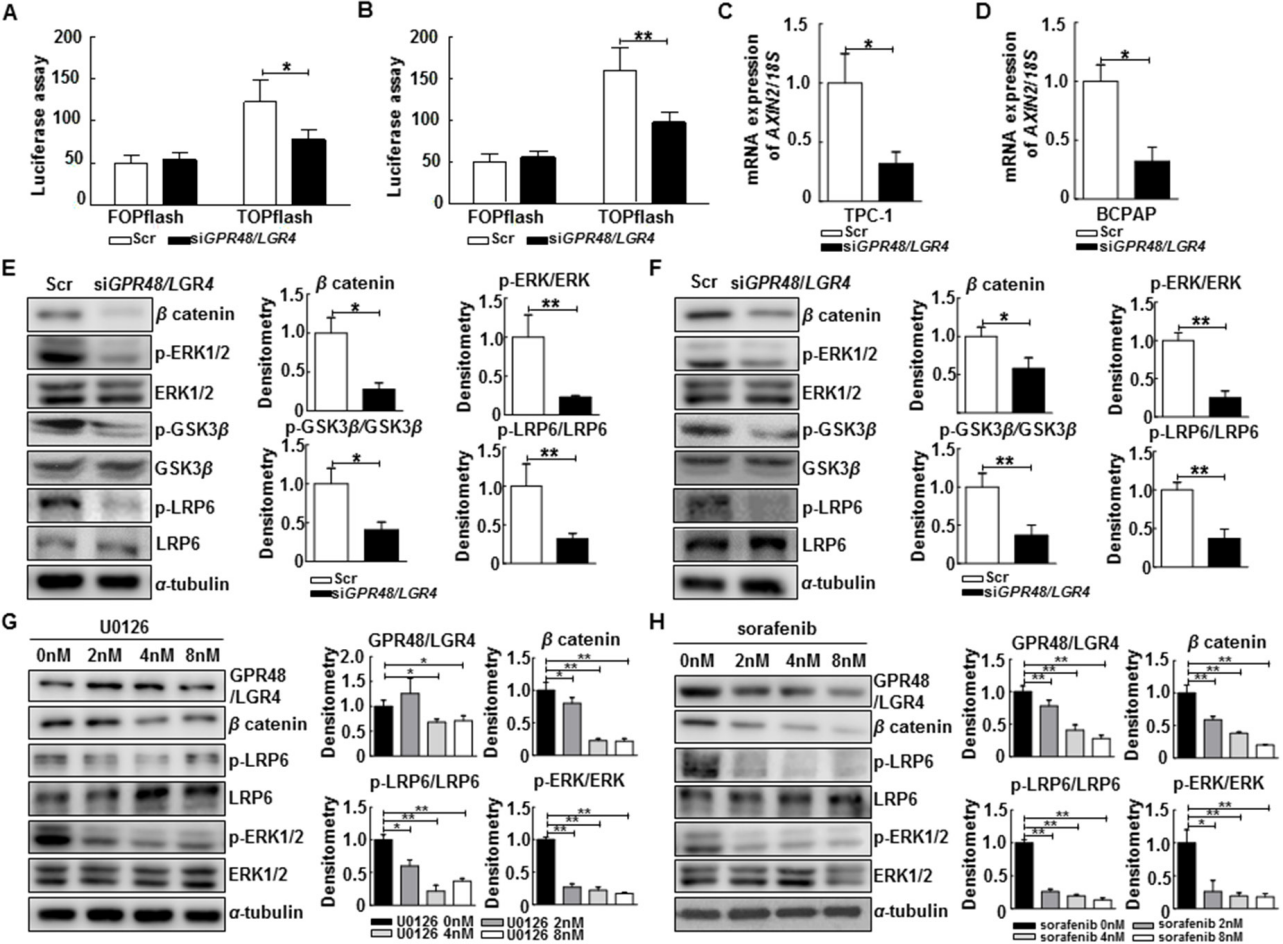
Regulation of the β-catenin pathway by GPR48/LGR4 via MAPK/ERK1/2 pathway **(A)** Effect of downregulation of GPR48/LGR4 on β-catenin signaling in TPC-1 using the luciferase-based TOPflash/FOPflash assay. **(B)** Effect of downregulation of GPR48/LGR4 on β-catenin signaling in BCPAP using the luciferase-based TOPflash/FOPflash assay. **(C)** *AXIN2* mRNA levels in TPC-1 cells transfected with *GPR48*/*LGR4*-specific or scrambled siRNA **(D)** *AXIN2* mRNA levels in BCPAP cells transfected with *GPR48*/*LGR4*-specific or scrambled siRNA. **(E)** Representative images of immunoblot analysis for detection of non-phospho β-catenin, phospho-ERK1/2, ERK1/2, phospho-GSK3β, GSK3β, phospho-LRP6, LRP6, and α-tubulin in TPC-1 cells transfected with *GPR48*/*LGR4*-specific or scrambled siRNA. **(F)** Representative images of immunoblot analysis for detection of of non-phospho β-catenin, phospho-ERK1/2, ERK1/2, phospho-GSK3β, GSK3β, phospho-LRP6, LRP6 and α-tubulin in BCPAP cells transfected with *GPR48*/*LGR4*-specific or scrambled siRNA. **(G)** Representative images of immunoblot analysis for detection of GPR48/LGR4, non-phospho β-catenin, phospho-LRP6, LRP6, phospho-ERK1/2, ERK1/2, and α-tubulin in TPC-1 cells treated with U0126 (MEK inhibitor). α-tubulin was used as a loading control. **(H)** Representative images of immunoblot analysis for detection of GPR48/LGR4, non-phospho β-catenin, phospho-LRP6, LRP6, phospho-ERK1/2, ERK1/2, and α-tubulin in BCPAP cells treated with sorafenib (RAF inhibitor). α-tubulin was used as a loading control. ‘Scr’ indicates cells transfected with scrambled siRNA. ^*^, *P*<0.05; ^**^, *P*<0.01.

GPR48/LGR4 activates the β-catenin signaling pathway and increases expression of its downstream target genes in human cancers [17, 18]. We investigated whether knockdown of *GPR48/LGR4* decreased β-catenin signaling in thyroid cancer cells using the luciferase-based TOPflash/FOPflash assay. *TCF4* transcription activity decreased 59.0% in TPC-1 cells and 56.1% in *GPR48/LGR4*-knockdown BCPAP cells in comparison with control cells (Figure 4A and 4B). In addition, we monitored expression of *AXIN2*, a major target gene of β-catenin, by RT-PCR. The level of *AXIN2* mRNA significantly decreased in both TPC-1 cells and *GPR48/LGR4*-knockdown BCPAP cells in comparison with control cells (Figure 4C and 4D). Moreover, protein expression levels of the non-phospho (active) β-catenin were much lower in cells transfected with *GPR48/LGR4* siRNA than in controls (Figure 4E and 4F). These results demonstrate that GPR48/LGR4 activates β-catenin signaling in differentiated thyroid carcinoma.

GPR48/LGR4 promotes epithelial cell proliferation and migration by activating the ERK and PI3K pathways [18, 23]. Activation of ERK induces phosphorylation of GSK3β on Ser9, resulting in stabilization of β-catenin [24, 25]. To determine the mechanism by which GPR48/LGR4 promotes thyroid tumorigenesis, we monitored the ERK1/2 and β-catenin pathways in *GPR48/LGR4*-knockdown thyroid cancer cells. In both TPC-1 and BCPAP cells, knockdown of *GPR48/LGR4* was associated with reductions in phosphorylation of LRP6, phosphorylation of GSK3β Ser9, and phosphorylation of ERK1/2, whereas total LRP6, total GSK3β and ERK1/2 levels were unchanged (Figure 4E and 4F). These data suggest that GPR48/LGR4 activates MAPK/ERK1/2 in PTC cells. To determine whether the MAPK/ERK1/2 pathway participates in regulation of GPR48/LGR4, we treated cells with pharmacological inhibitors of RAF (sorafenib) or MEK (U0126) and subjected them to immunoblot analysis. Treatment of TPC-1 cells with U0126 markedly decreased the phosphorylation levels of LRP6 and the level of non-phospho (active) β-catenin. Treatment of BCPAP cells with sorafenib also markedly decreased the expression of GPR48/LGR4 and activation of the β-catenin pathway (Figure 4G and 4H). Neither inhibitor affected the expression of *GPR48/LGR4* mRNA (Supplementary Figure 2). These results suggest that the MAPK/ERK1/2 pathway is required for GPR48/LGR48-mediated β-catenin signaling, and that post-transcriptional regulation of GPR48/LGR4 in differentiated thyroid carcinoma may be influenced by inhibition of RAF and MEK.

### The RSPO2–GPR48/LGR4 signaling axis in PTC

**Figure 5:**
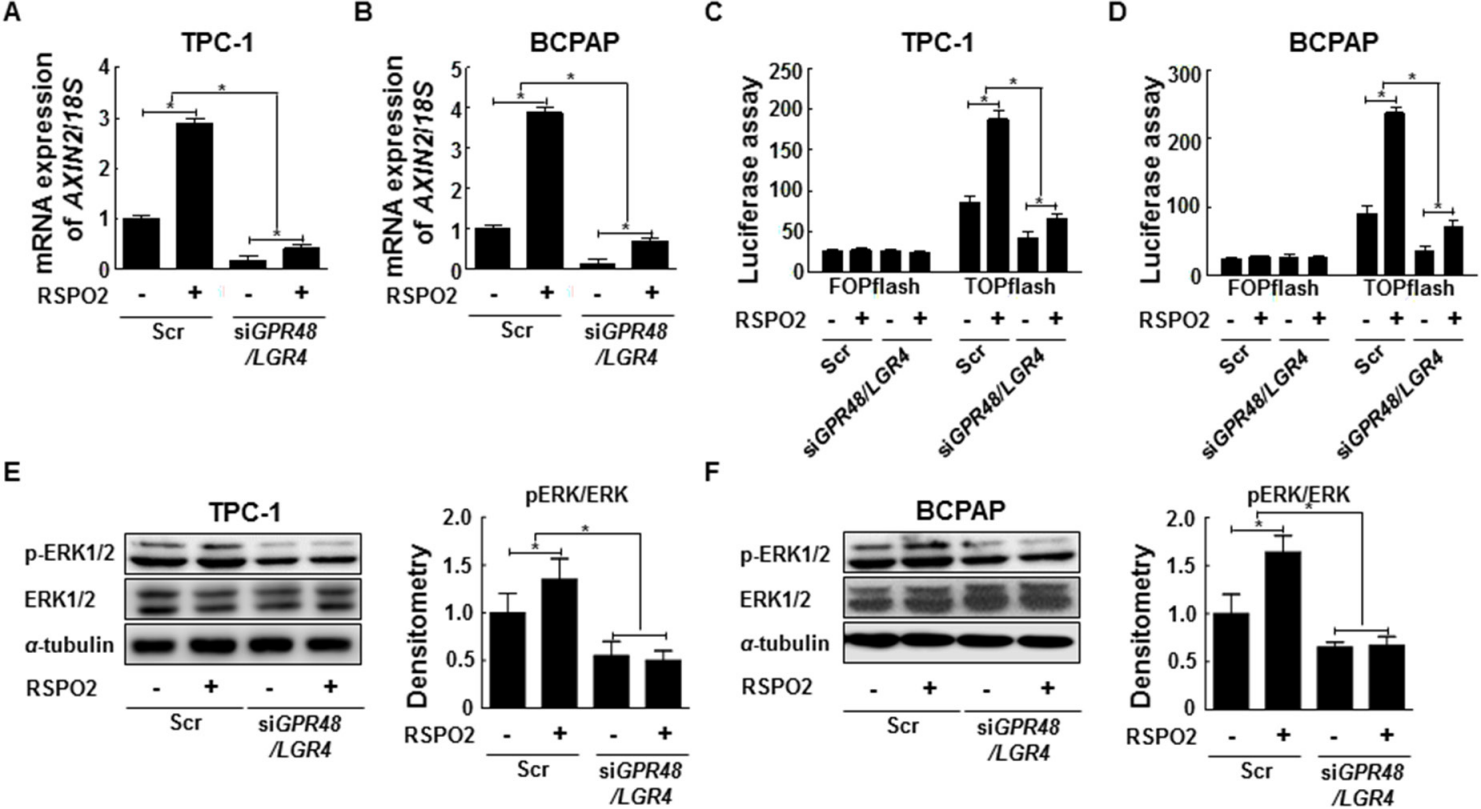
RSPO2-GPR48/LGR4 signaling axis in papillary thyroid carcinoma **(A)** *AXIN2* mRNA levels in TPC-1 cells treated with RSPO2 (100 ng/mL) for 24 hr after transfection with *GPR48*/*LGR4*-specific or scrambled siRNA. **(B)** *AXIN2* mRNA levels in BCPAP cells treated with RSPO2 (100 ng/ml) for 24 hr after transfection with *GPR48*/*LGR4*-specific or scrambled siRNA. **(C)** β-catenin signaling in TPC-1 cells transfected with *Gpr48/Lgr4*-specific or scrambled siRNA in response to treatment with RSPO2 (100 ng/mL), as determined by the luciferase-based TOPflash/FOPflash assay. **(D)** β-catenin signaling in BCPAP cells transfected with *GPR48/LGR4*-specific or scrambled siRNA in response to treatment with RSPO2 (100 ng/mL), as determined by the luciferase-based TOPflash/FOPflash assay. **(E)** Representative images of immunoblot analysis for detection of ERK1/2, phospho-ERK1/2, and α-tubulin in TPC-1 cells treated with RSPO2 (100 ng/mL) for 24 hr after transfection with *GPR48*/*LGR4*-specific or scrambled siRNA. α-tubulin was used as a loading control. **(F)** Representative images of immunoblot analysis for detection of ERK1/2, phospho-ERK1/2, and α-tubulin in BCPAP cells treated with RSPO2 (100 ng/mL) for 24 hr after transfection with *GPR48*/*LGR4*-specific or scrambled siRNA. α-tubulin was used as a loading control. ‘Scr’ indicates cells transfected with scrambled siRNA. ^*^, *P* < 0.05.

RSPOs promote activation of the Wnt pathway by binding LGRs [11, 26]. Based on this premise, we tested whether exogenous RSPO2 could directly increase β-catenin signaling, using a reporter assay and monitoring *AXIN2* expression in control and *GPR48/LGR4*-knockdown thyroid cancer cells. Previous studies report that the functional effects of RSPOs via GPR48/LGR4 are maximal in Wnt3A-conditioned media. Therefore, we harvested cells with Wnt3A-conditioned media and investigated the change in β-catenin signaling in cancer cells treated with RSPO2 [11, 26]. TCP-1 and BCPAP cells were treated with RSPO2 (100 ng/mL) for 24 hr after 48-hr transfection with *GPR48/LGR4*-specific or scrambled siRNA. Expression of *AXIN2* and *TCF4* transcriptional activity increased in both control and *GPR48/LGR4*-knockdown cells after treatment with exogenous RSPO2 (Figure 5A–5D).

To investigate the effect of RSPO2 on the β-catenin pathway in relation to GPR48/LGR4, we compared the fold change of *AXIN2* and *TCF4* transcriptional activity following RSPO2 treatment between *GPR48/LGR4*-knockdown and control cells. Knockdown cells treated with RSPO2 treatment exhibited reductions in the fold change of *AXIN2* expression and *TCF4* transcriptional activity in comparison with control cells (Figure 5). We also investigated the RSPO2–GPR48/LGR4 signaling axis in Nthy-ori3-1 cells, which express lower endogenous levels of *RSPO2* than cancer cells. In normal thyroid cells, exogenous RSPO2 treatment also induced activation of the β-catenin signaling pathway (Supplementary Figure 3). These findings suggest that the RSPO2–GPR48/LGR4 signaling axis may be important in normal thyroid physiology, as well as in thyroid tumorigenesis.

To investigate the regulation of ERK1/2 phosphorylation by RSPO2–GPR48/LGR4 signaling axis, we monitored ERK1/2 phosphorylation in *GPR48/LGR4*-knockdown and control cells treated with exogenous RSPO2. ERK1/2 phosphorylation significantly increased in control BCPAP and TPC1 cells upon RSPO2 treatment (Figure 5E, and 5F), but not in *GPR48/LGR4*-knockdown cells. Based on these findings, we speculate that exogenous RSPO2 treatment can induce increased ERK1/2 phosphorylation in thyroid cancer cells, and that the effect of RSPO2 is strongly dependent on expression of GPR48/LGR4.

## DISCUSSION

In this study, we obtained clear evidence of the functional expression of GPR48/LGR4 in thyroid cancer cells *in vitro* and *in vivo*. Furthermore, we found that RSPO2, a ligand of GPR48/LGR4, was expressed at higher levels in PTC, and that it activated β-catenin signaling via GPR48/LGR4. These observations suggest that elevated activity of the RSPO2–GPR48/LGR4 signaling axis in tumor cells determines the behaviors of thyroid cancers. This idea is further supported by the elevated viability and migration of tumor cells expressing higher levels of GPR48/LGR4. More importantly, the clinical observations revealed that patients with higher expression of GPR48/LGR4 tended to experience more rapid regional tumor progression, including metastasis. These findings provide the first evidence that the RSPO2–GPR48/LGR4 pathway is hyperactivated in thyroid cancer, particularly PTC, and acts as a tumor-promoting factor.

LGRs were originally identified in *in silico* screens for cDNAs encoding proteins with homology to the glycoprotein hormone receptor class of G-protein-coupled receptors (GPRs) such as TSH [5, 6, 27]. Early studies showed that GPR48/LGR4 and GPR49/LGR5 are closely related, as reflected by the similarities in their amino acid sequences; however, *GPR48/LGR4* is the only transcript found in rodent thyroid gland [7]. In this study, we obtained consistent results in human thyroid gland, which exhibited focal and low levels of GPR48/LGR4 expression. Recent efforts elucidated the role of GPR48/LGR4 and GPR49/LGR5 by demonstrating that these receptors are the signaling platform for epithelial stem cells [28, 29]. Although we detected focal and low levels of GPR48/LGR4 expression, the functional role of this factor in epithelial homeostasis in adult thyroid gland remains to be determined.

Recently, several groups revealed that the RSPOs, which are secreted Wnt agonists, are the endogenous ligands of GPR48/LGR4 and GPR49/LGR5, revealing the crucial role of LGR proteins in epithelial stem cell homeostasis in the intestines [11, 26, 30, 31]. Subsequent studies revealed that the RSPO–GPR48/LGR4 and GPR49/LGR5 ligand receptor system constitute a major axis involved in regulation of Wnt signaling, reflected by its pleiotropic roles in development, survival of adult stem cells, and carcinogenesis [11, 32]. More recent work showed that RSPOs potentiate Wnt/β-catenin signaling through GPR48/LGR4 [26, 31, 33]. In the presence of RSPOs and LGR receptors, the Frizzled receptor is activated, leading to repression of GSK3β, accumulation of active β-catenin, and its translocation to the nucleus [34]. Additionally, multiple studies demonstrated that in the context of tumorigenesis, GPR48/LGR4 activates Wnt/β-catenin signaling via the AKT and ERK pathway. In response to certain growth stimuli and oncogenes, activated AKT and ERK1/2 can phosphorylate Ser9 of GSK3β, resulting in activation of Wnt/β-catenin signaling during tumorigenesis [24, 25]. Overexpression of GPR48/LGR4 increases phosphorylation levels of AKT and ERK1/2, and GPR48/LGR4 activates Wnt/β-catenin signaling via a PI3K- and ERK1/2-dependent mechanism in colon carcinogenesis [16, 17]. Furthermore, inhibition of GPR48/LGR4 is associated with downregulation of AKT and the mTOR signaling pathway in prostate tumorigenesis [18]. To date, however, few studies have investigated the function of RSPOs and GPR48/LGR4 in thyroid tumorigenesis.

Aberrant activation of Wnt/β-catenin signaling is strongly associated with thyroid tumorigenesis [35]. Previous studies reported expression of components of the Wnt signaling pathway, including Wnt factors, members of the Frizzled receptor family, and Disheveled isoforms, in human thyroid cells [36]. In addition, these studies revealed the existence of a degradation complex consisting of APC, Axin, and GSK3β in human thyroid cells. In the context of thyroid tumorigenesis, Ishigaki et al., reported that β-catenin levels in human PTC are significantly correlated with cyclin D1 expression [35]. Recent studies report that inhibition of β-catenin diminishes tumorigenesis of differentiated thyroid cancer [19, 37]. In addition, the proto-oncogene RET/PTC induces nuclear accumulation of β-catenin in PTC by activating the PI3K/AKT and MAPK signaling pathways, but the precise mechanism underlying β-catenin dysregulation in differentiated thyroid carcinoma remains unknown [38, 39].

We detected an association between GPR48/LGR4 expression and diverse clinicopathologic findings in PTC, and also observed a significant association between GPR48/LGR4 expression and the presence of the BRAF mutation in multivariate analysis. Knockdown of *GPR48/LGR4* in tumor cells was significantly associated with reduced phosphorylation of Ser9 of GSK3β and ERK1/2, resulting in downregulation of β-catenin signaling. Elucidation of the precise mechanism by which GPR48/LGR4 influences the ERK pathway will require additional experiments, e.g., immunoprecipitations and enzymatic binding assays.

Collectively, our observations support the idea that aberrant increase of GPR48/LGR4–β-catenin pathway promotes thyroid tumorigenesis through the RAF–RAS pathway. Consistent with this, administration of RSPO1 to mice increases cell size in the small intestine, and RSPO fusions occur in colon and lung cancer [40–41], [32]. In this study, we demonstrated that exogenous RSPO2 treatment increased GPR48/LGR4-dependent ERK activation in thyroid cancer cells.

Accordingly, our results suggest that GPR48/LGR4 protein is worth studying as a new therapeutic target in PTC. Notably in this regard, a recent study reported an association between GPR49/LGR5 and human PTC aggressiveness [42]. Overexpression of LGR5 in human PTC is associated with several markers of tumor aggressiveness and predicted LN metastasis. Consistent with this, we observed that GPR49/LGR5 was overexpressed in thyroid tumor tissue relative to normal tissue, although there was no significant association between GPR49/LGR5 and clinicopathologic parameters (Supplementary Table 1).

In summary, we showed that the RSPO2 and GPR48/LGR4 function in thyroid tumorigenesis, particularly in patients with regional tumor progression including LN metastasis. These effects may involve the intrinsic stimulatory role of the RSPO2–GPR48/LGR4 signaling axis on proliferation and migration of thyroid cancer cells. Our findings suggest that GPR48/LGR4 represents a novel target for treating differentiated thyroid carcinoma by modulating the β-catenin pathway.

## MATERIALS AND METHODS

### Patients and tissue samples

Data were retrospectively analyzed from 214 patients with PTC who underwent total thyroidectomy and cervical LN dissection at Chungnam National University Hospital from 2003 to 2010. Prophylactic central LN dissection was performed in 148 patients without clinical evidence of positive LN on imaging or palpation. Twenty-five patients with clinically evident positive central LN underwent therapeutic central LN dissection. The remaining 41 patients underwent central and lateral LN dissection due to evidence of metastatic LN in the lateral neck before surgery. Lateral LN dissection was performed using a modified radical operation that involved complete removal of level II-V lateral cervical LNs. Level I dissection was not performed without clinical evidence of metastases at level I. Patients who underwent lobectomy only, but not central LN dissection, or whose medical record was unclear, were excluded from the study. All specimens were collected from patients after informed consent was obtained in accordance with the institutional guidelines of Chungnam National University Hospital. Diagnosis was made according to the World Health Organization classification of endocrine organ tumors [43]. Surveillance for recurrent disease usually consisted of physical examination, serum thyroglobulin (Tg) level, and ultrasonography every 12 months for up to 5 years, and patients were followed for a mean duration of 86.6 ± 22.5 months to evaluate tumor recurrence [44].

### Immunohistochemistry

Tissues were retrieved from the archives of the Department of Pathology, Chungnam National University Hospital, South Korea. Before immunohistochemistry, 4-micron–thick sections of paraffin-embedded tissue blocks were incubated at 56°C for 3 hr. Specimens were stained using the Vectastain ABC HRP kit (Vector Laboratories, Inc., Burlingame, CA, USA). Antigen retrieval was performed by microwaving in citrate buffer for 10 minutes. Endogenous peroxidase activity was inactivated by incubation in 3% hydrogen peroxide for 10 minutes. Non-specific binding sites were blocked by incubation in 10% normal goat serum in phosphate-buffered saline (PBS). Tissue section slides were incubated for 1 hr at room temperature with primary antibodies against GPR48/LGR4 (1:50, Atlas Antibodies, Stockholm, Sweden) and BRAF^V600E^ (Beecher Instruments, Silver Spring, Maryland) as described [45, 46]. Tissue sections were counterstained with hematoxylin. Negative controls were incubated with PBS instead of primary antibody, and no positive staining was observed. In addition, positive controls were performed with sections of colon adenocarcinoma and stained for GPR48/LGR4. Tissue slides were analyzed on an OLYMPUS BX51 microscope. Microscopic analysis was performed by two certified pathologists who were unaware of the identity of the samples and the corresponding clinicopathologic data. To quantify GPR48/LGR4 and BRAF^V600E^ IHC staining, a scoring system was used that combined the intensity and distribution of positive staining: 0, no staining; +1, weak staining in focal tumor areas; +2, moderate staining in most tumors; and +3, strong staining in most tumors. Finally, for statistical comparison, tissue slides with scores of 0, +1, or +2 were included in the low-GPR48/LGR4 immunoexpression group, and those with a score of +3 were included in the high-GPR48/LGR4 immunoexpression group. Specimens with no staining for BRAF^V600E^ were classified as lacking the BRAF^V600E^mutation, and those with scores of +1, +2, or +3 for BRAF^V600E^ immunoexpression were classified as having the BRAF^V600E^mutation.

### Cell lines

The human anaplastic thyroid cancer cell line, 8505C, was purchased from DSMZ (Braunschweig, Germany). Nthy-ori 3-1, a normal thyroid follicular cell line from human adult, was obtained from Dr. Anna Maria Porcelli (Bologna University, Bologna, Italy). The human PTC cell lines BCPAP and TPC-1 were provided by Dr. M. Santoro (Università di Napoli Federico II, Naples, Italy) and Dr. Masahide Takahashi (Nagoya University, Nagoya, Japan), respectively.

### Cell culture and transfection

BCPAP and TPC-1 were maintained in Dulbecco’s Modified Eagle’s Medium (DMEM; Invitrogen) [47–49]. Nthy-ori3-1 and 8505C were cultured in RPMI 1640. Both types of media were supplemented with 10% heat-inactivated fetal bovine serum (FBS), 100 U/mL penicillin, and 100 μg/mL streptomycin [47, 48]. Cells were cultured at 37°C in a humidified chamber under a 5% CO_2_ atmosphere. Transient transfection was performed using the Lipofectamine 2000 reagent (Invitrogen, Carlsbad, CA, USA) when cells reached 60% confluence. The full-length cDNA encoding human GPR48/LGR4 was PCR-amplified from a cDNA library and inserted into the pCMV6-Entry (cat #PS100001) vector purchased from OriGene (Rockville, MD, USA). TPC-1 and BCPAP cells were transfected with 20 pmol Stealth siRNA or si*GPR48/LGR4* (Invitrogen, Carlsbad, CA, USA) oligomers in 50 μL Opti-MEM I using Lipofectamine RNAiMAX (Invitrogen, Carlsbad, CA, USA). All experiments were performed in duplicate and repeated at least three times.

### Recombinant human RSPO2 treatment in human thyroid cell lines

The immortalized normal thyroid cell line, Nthy-ori3-1, and thyroid cancer cell lines BCPAP and TPC-1 were treated with recombinant human RSPO2 (RSPO2, R&D Systems, 3266-RS-025). Recombinant RSPO2 was diluted to 100 μg/mL in PBS containing 0.1% bovine serum albumin. Human cell lines were treated with RSPO2 at 100 ng/mL, harvested after 24 hr in Wnt3A-conditioned media, and then processed for mRNA, protein, and luciferase assays. To exclude the effect of bovine serum albumin, control cells were treated with 0.1% bovine serum albumin.

### Total RNA isolation and quantitative real-time reverse transcriptase-polymerase chain reaction (RT-PCR)

Total RNA was isolated using Trizol (Invitrogen, Carlsbad, CA, USA). cDNA was prepared from total RNA using M-MLV Reverse Transcriptase and oligo-dT primers (Invitrogen, Carlsbad, CA, USA). Real-time PCR was performed using cDNA, QuantiTect SYBR Green PCR Master Mix (QIAGEN), and specific primers (Supplementary Table 2). PCR conditions were as follows: 40 cycles of 95°C for 15 seconds, 60°C for 1 minute, and 72°C for 1 minute [47, 48, 50, 51].

### Western blot analysis

Cells were washed twice in ice-cold PBS. Whole-cell protein extracts were obtained by lysis with sodium dodecyl sulfate–polyacrylamide gel electrophoresis (SDS-PAGE) sample buffer [62.5mMTris-HCl (PH 6.8), 2% SDS, 10% glycerol, 50 mM dithiothreitol (DTT), 0.01% bromophenol blue, protease inhibitors]. Protein concentrations in cell lysates were measured using the Bradford reagent (Bio-Rad). Prior to loading, samples were denatured by boiling for 10 minutes, and then separated by SDS-PAGE on 5–10% gels. Resolved proteins were transferred to nitrocellulose (NC) (Amersham Biosciences, Germany) or polyvinylidene difluoride (PVDF) membrane (Thermo Fisher Scientific, USA). The membranes were blocked for 2 hr in Tris-buffered saline (TBS) containing 0.1% Tween 20 (Sigma Aldrich) and 3% BSA. The blots were then incubated overnight at 4°C with primary antibodies against GPR48/LGR4 (Abcam), GAPDH (Cell Signaling Technology), ERK (Cell Signaling Technology), phospho-ERK (Cell Signaling Technology), GSK3β (Cell Signaling Technology), phospho-GSK3β (Cell Signaling Technology), non-phospho β-catenin (Cell Signaling Technology), phospho-LRP6 (Cell Signaling Technology), LRP6 (Cell Signaling Technology), and α-tubulin (Abcam). The blots were washed three times for 10 minutes each with TBS containing 0.1% Tween-20. Immunoreactive bands were developed using peroxidase-conjugated secondary antibodies (Phototope-HRP Western Blot Detection Kit; New England Biolabs, Beverly, MA, USA).

### Cell viability assay

The cells were seeded at 5.0 × 10^3^ cells/mL in 96-well microplates and allowed to attach for 24 hr. After transfection with control, *GPR48/LGR4* cDNA, siRNA, si*GPR48/LGR4* for 48 hr, cell viability was assessed using the Cell Counting Kit-8 (CCK-8, Dojindo Molecular Technologies, Maryland, USA). Briefly, the highly water-soluble tetrazolium salt WST-8 [2-(2-methoxy-4-nitrophenyl)-3-(4-nitrophenyl)-5-(2, 4-disulfophenyl)-2H-tetrazolium, monosodium salt] produces an orange-colored water-soluble product, formazan. The amount of formazan dye generated by dehydrogenases in cells is directly proportional to the number of living cells. CCK-8 (10 mL) was added to each well and incubated for 3 hr at 37°C, and then cell viability was assessed by measuring absorbance at 450 nm on a microplate reader. Three replicate wells were used for each experimental condition.

### Cell migration assay

Migration of PTC cells was performed using Transwell chambers (Corning Costar, Cambridge, MA) with polycarbonate filters 6.5 mm in diameter (8 μm pore size). For the migration assay, the lower surface of the filter was coated with 10 μg of gelatin, and fresh medium containing 5% FBS was placed in the lower wells. PTC cells (BCPAP or TPC-1) were transfected with cDNA or siRNA for 48 hr, incubated for 24 hr in medium containing 1% FBS, trypsinized, and suspended at a final concentration of 1 × 10^6^ cells/mL in medium containing 1% FBS. Cell suspension (100 μL) was loaded into each of the upper wells, and the chambers were incubated at 37°C for 6 hr. Cells were fixed and stained with crystal violet. Non-migrating cells on the upper surface of the filter were removed by wiping with a cotton swab. Chemotaxis was quantified by counting the cells that migrated to the lower side of the filter, using an optical microscope (200×); eight randomly chosen fields were counted for each migration assay [52].

### TOPflash/FOPflash luciferase assay

To assess β-catenin activity induced by GPR48/LGR4, thyroid cancer cells (BCPAP and TPC-1) were treated with si-*GPR48/LGR4* or si-control for 48 hr, and then transiently transfected for 24 hr with TOPflash and FOPflash plasmids (2 μg each) provided by Dr. Dae-Sik Lim (KAIST, Korea) using Lipofectamine Plus (Invitrogen) and OptiMEM (Invitrogen). Luciferase levels were determined using a luciferase assay kit (Promega). For each sample, the TOPflash reporter activity is presented as the relative ratio of normalized against *Renilla* luciferase activity. All experiments were performed in triplicate.

### Statistical analysis

Results are shown as means ± standard deviation (SD). Fisher’s exact test and two-tailed t-test were used to compare patients’ clinicopathologic data. Patients were divided into two groups, high- and low-immunostaining, according to GPR48/LGR4 expression as described above. Group comparisons of categorical variables were performed by linear-by-linear association and multivariate analysis using a stepwise logistic. All *in vitro* experiments were repeated three times, and statistical significance was analyzed using two-tailed Student’s t-test or one-way analysis of variance (ANOVA) followed by Tukey’s post hoc test. Data are presented as means ± SD, and a *P* value < 0.05 was considered statistically significant (^*^P< 0.05; ^**^P< 0.01). The SPSS software (Version 20) was used for all statistical analyses.

## SUPPLEMENTARY MATERIALS FIGURES AND TABLES



## Abbreviations

GPCR: G-protein coupled receptor
LGR: leucine-rich repeat–containing G-protein-coupled receptor
GPR48: G-protein coupled receptor 48
RSPO: R-spondin
LRP6: Low-density lipoprotein receptor–related protein 6
P27^kip1^: Cyclin-dependent kinase inhibitor 1B
TCF4: T cell transcription factor 4
PI3K: phosphoinositide 3-kinase
AKT: protein kinase B
PTC: papillary thyroid carcinoma
SCNs: solid cell nests
Nkx2.1: NK2 homeobox 1
RT-PCR: reverse transcription polymerase chain reaction
US: ultrasonography
FNAB: fine needle aspiration biopsy
LN: lymph node
GSK3β: glycogen synthase kinase 3 beta
ERK1/2: extracellular signal–regulated kinases
½ TSH: thyroid-stimulating hormone
APC: adenomatous polyposis coli
