# Supplementary material for: Upregulation of RSPO2-GPR48/LGR4 signaling in papillary thyroid carcinoma contributes to tumor progression
Source: Oncotarget. 2017 Nov 25;8(70):114980–94. doi: 10.18632/oncotarget.22692 (PMC5777747; doi:10.18632/oncotarget.22692)
Supplement: Supplementary file 1 [file oncotarget-08-114980-s001.pdf]

Upregulation of RSPO2-GPR48/LGR4 signaling in papillary thyroid carcinoma contributes to tumor progression

SUPPLEMENTARY MATERIALS

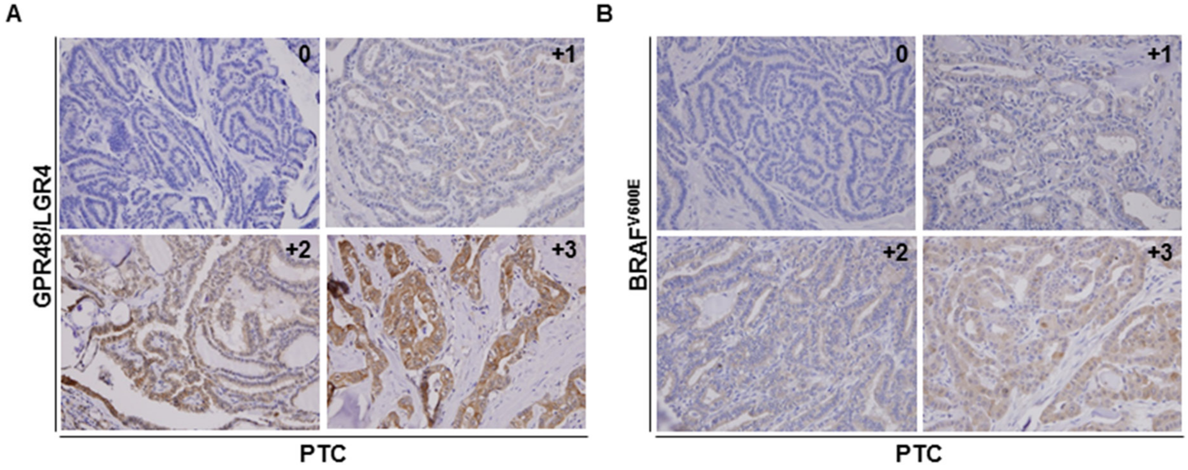

Supplementary Figure 1: Immunohistochemical evaluation of GPR48/LGR4 and BRAFV^{600E} expression in PTC tissue. **(A)** Representative immunohistochemical images of GPR48/LGR4. **(B)** Representative immunohistochemical images of BRAF^{V600E}. 0, no staining intensity; +1, weak staining intensity; +2, moderate staining intensity; +3 strong staining intensity. Magnification : 100×

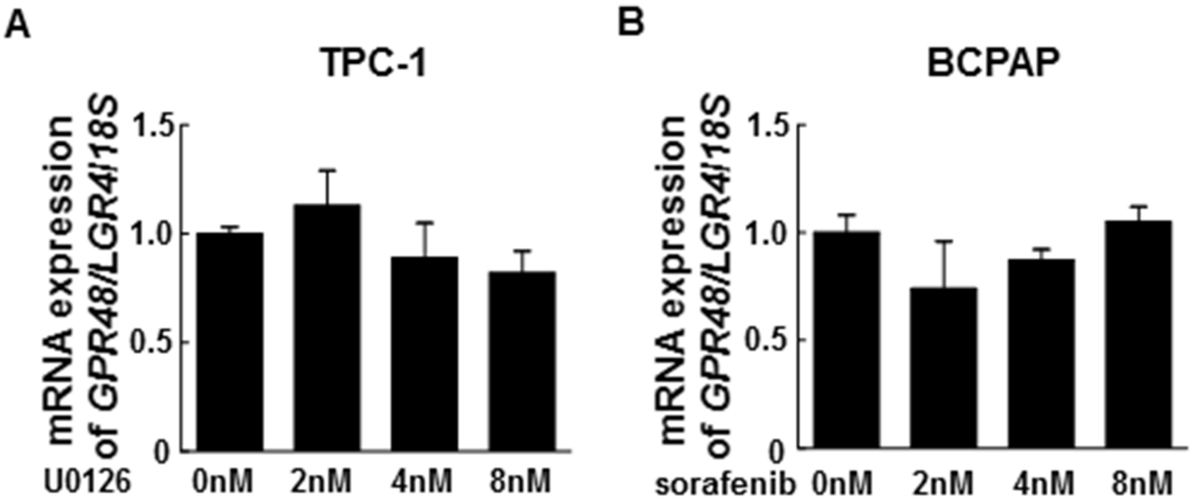

Supplementary Figure 2: *GPR48/LGR4* mRNA levels in total cell lysates of thyroid cancer cells treated with MEK or RAF inhibitor. **(A)** *GPR48/LGR4* mRNA levels in total cell lysates of TPC-1 cells treated with U0126 (MEK inhibitor) treatments, as determined by RT-PCR. **(B)** *GPR48/LGR4* mRNA levels in total cell lysates in BCPAP cells treated with sorafenib (RAF inhibitor), as determined by RT-PCR.

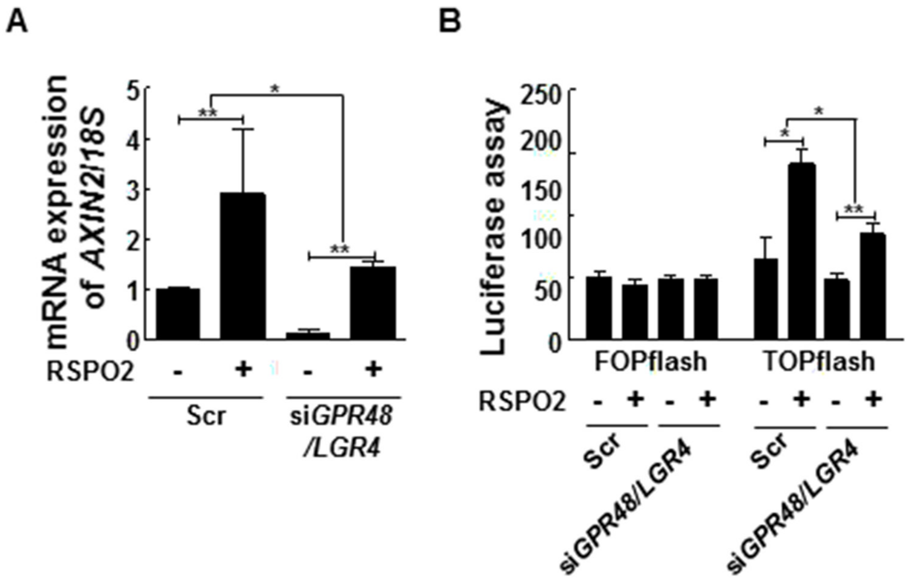

Supplementary Figure 3: RSPO2–GPR48/LGR4 signaling axis in human normal thyroid cells. (A) *AXIN2* mRNA levels in Nthy-ori3-1 cells treated with RSPO2 (100 ng/mL) for 24 hr after transfection with *GPR48/LGR4*-specific or scrambled siRNA. **(B)** β-catenin signaling in Nthy-ori3-1 cells transfected with *GPR48/LGR4*-specific or scrambled siRNA in response to treatment with RSPO2 (100 ng/mL), as determined by the luciferase-based TOPflash/FOPflash assay. *, $P<0.05$; **, $P<0.01$.

Supplementary Table 1: Relationship between intensity of GPR49/LGR5 staining and clinicopathologic factors in 214 patients

Variables		No. of patients	LGR5		
			Low	High	*P* value
Age (years)	< 45<br>≥ 45	81<br>133	47<br>74	34<br>59	0.733
Gender	Male<br>Female	39<br>175	26<br>95	13<br>80	0.158
Tumor size	≤ 1cm<br>> 1cm	85<br>129	46<br>75	39<br>54	0.561
Multicentricity	No<br>Yes	125<br>89	75<br>46	50<br>43	0.227
Microscopic capsular invasion	No<br>Yes	52<br>162	31<br>90	21<br>72	0.607
Extrathryoid extension	No<br>Yes	64<br>150	37<br>84	27<br>66	0.807
Lymphovascular invasion	No<br>Yes	43<br>171	23<br>98	20<br>73	0.651
Lymph node metastasis	No<br>Yes	79<br>135	50<br>71	29<br>64	0.128
Central Lymph node metastasis	No<br>Yes	79<br>135	50<br>71	29<br>64	0.128
Lateral Lymph node metastasis	No<br>Yes	173<br>41	96<br>25	77<br>16	0.524
Locoregional recurrence	No<br>Yes	198<br>16	111<br>10	87<br>6	0.617
$BRAF^{V600E}$ mutation	No<br>Yes	34<br>180	19<br>102	15<br>78	0.933

Supplementary Table 2: Primer pairs used for mRNA determinations

	Sense primer	Antisense primer
GPR48/LGR4	AGGTGGTTGTCTGGAACA	AAGAAACGATTCGCAGCAGT
RSPO2	CACATTCCATGGTTTCCTCT	CAAGTGCAAAGTAGGCTTTT
RSPO3	GACAGCAAAAGTCTGGAATC	CCGATTTCTGTTTATCTTGG
AXIN2	AGTGTGAGGTCCACGGAAAC	ACAGGATCGCTCTTGAA
18S	AGGAATTCCCAGTAAGTGCG	GCCTCACTAAACCATCCAA
